# Effects of imposed and self-selected exercise on perceptual and affective responses, muscle function, quality, and functionality of strength training in older women and men: a randomized trial

**DOI:** 10.1590/1414-431X2024e13968

**Published:** 2024-12-02

**Authors:** E.D.S.A. Garcia, S.S. Ferreira, R. Lazzarotto, J.K.F. da Silva, P.C.B. Bento

**Affiliations:** 1 Departamento de Educação Física, Universidade Federal do Paraná, Curitiba, PR, Brasil; 2 Instituto Federal do Espírito Santo, Piúma, ES, Brasil

**Keywords:** Affect, Rated perceived exertion, Strength training, Self-selected, Elderly

## Abstract

The objective of the present randomized trial was to verify the effect of twelve weeks of strength training with self-selected and imposed loads on muscle function, functionality, muscle quality, and perceptual and affective responses in elderly men and women. Twenty-four volunteers were divided into two groups of 12 individuals each: self-selected group (SS) (8 women, 4 men; mean age=66.92±6.18 years) and imposed group (IMP) (8 women, 4 men; mean age=65.33±2.42 years). The strength exercise program lasted 12 weeks (3 d/w). All exercises were performed on machines. The SS group was instructed to select a weight that would allow them to complete three sets of 10 repetitions, while the IMP group had the load imposed by the researchers following the exercise prescription model recommended by American College of Sports Medicine (ACSM). Rated perceived exertion (RPE) and affective responses were recorded at the end of each session. Muscle function, functionality, and muscle quality were assessed before and after the intervention. Both groups demonstrated similar improvements in strength and functional capacity. Furthermore, the SS group reported lower RPE and higher affective responses compared to the IMP group at 8-12 weeks. In summary, the findings from this study highlighted the effectiveness of both IMP and SS intensity resistance training programs in enhancing muscle strength and functional capacity among older adults.

## Introduction

Strength training is one of the main non-pharmacological strategies to support the human aging process (1). This type of exercise not only improves mobility, physical functionality, and performance in daily activities, but also plays a crucial role in preserving the independence of the elderly, among other benefits (2).

Various strength training variables, including the number of sets, load, rest time, exercise type, and training methods (3,4), are often manipulated as a strategy to promote neuromuscular adaptations to achieve improvements in muscle hypertrophy, strength, and power (4). However, the manipulation of these variables can also influence behavioral and psychological aspects, impacting individual perceptions of exercise practice (5).

Research indicates that even minimal strength training (with 3 exercises per session, ≤60 min weekly across 2 days) can result in psychophysiological benefits (1,6). Nevertheless, it is a significant challenge for healthcare professionals to encourage engagement and adherence to these exercise regimens, especially among older individuals.

Elderly individuals often have poor adherence to strength training programs, leading to significant dropout rates (7). As outlined by Sperandei et al. (7), approximately 63% of new adult participants who initiate an exercise program cease the activity within the initial 3 months, with only 4% maintaining their commitment for more than 12 months. The authors emphasize that the correlation between physiological aspects (such as weight loss, hypertrophy, etc.) and psychological factors such as motivation, along with other variables, contributes significantly to this dropout trend.

Scientific research in recent decades shows that exercise prescription, combining psychological and physiological factors, has been thoroughly investigated (8). Within this framework, self-selected (SS) intensity has emerged as a way to enhance the individual sense of autonomy and increase enjoyment during and after exercise sessions (9). While research on SS intensity has primarily focused on aerobic exercises, it has only been explored to a limited extent in strength training among the elderly population.

The findings from Elsangedy et al. (10) revealed that the intensity chosen by elderly individuals themselves was aligned with the American College of Sports Medicine (ACSM) guidelines for deconditioned and novice elderly individuals in strength training. In a subsequent intervention conducted by Elsangedy et al. (9) in 2021, a 12-week training program with SS intensity not only resulted in improvements in neuromuscular parameters and cardiorespiratory fitness but also triggered positive emotional responses. Furthermore, Herda et al. (11) demonstrated that a 12-week training protocol with SS intensity involving both strength and aerobic exercises, improved neuromuscular and physiological aspects among the elderly, irrespective of the utilization of dietary supplements like whey protein.

Although short-term intervention studies show that training with SS intensity provides psychophysiological benefits (9-11), there are no studies that have analyzed the effect of an SS intensity exercise program compared to traditional exercise-imposed prescription concerning muscular function, functionality, muscle quality, and perceptual and affective aspects in the elderly. Therefore, the purpose of this study was to examine and compare the impact of twelve weeks of strength training with imposed and self-selected loads on muscular function, functionality, muscle quality, and perceptual and affective responses in the elderly.

The outcomes of this study will contribute to a deeper understanding of the psychophysiological aspects involved in imposed and self-selected prescription exercises. Additionally, it will help the prescription and monitoring of strength training among the elderly.

## Material and Methods

### Study design

The study was a quasi-experimental randomized clinical trial. All testing procedures were conducted at CECOM (Center for Motor Behavior Studies) at the Federal University of Paraná between June 2022 and December 2022. This study was approved by the Ethics Committee of the Federal University of Paraná under number 5.219.713 and registered in the Brazilian Clinical Trials Registry (REBEC) under number U1111-1276-0642.

### Sample size

The sample size was calculated using G*Power 3.1 software, considering the following parameters: i) linear mixed model test; ii) 95% confidence level; iii) 5% sample error; iv) 80% analysis power; v) number of groups=2; vi) number of measurements=2 (pre- and post-experiment assessment); and vii) an additional 10% to compensate for possible losses and refusals. Therefore, the initially estimated sample was 30 elderly individuals divided into two groups. After obtaining the informed consent from the participants, a stratified randomization was adopted based on the participants' levels of physical activity using IPAQ (International Physical Activity Questionnaire), and the participants were then allocated into two blocks of equal numbers of men and women. The randomization process was made using the Software Jamovi version 2.3.9. The data distribution was tested using boxplots and the Shapiro-Wilk test. A linear mixed model was used to assess the within- and between-group effects for each outcome. In the case of a significant F-value, Bonferroni *post hoc* tests were employed to identify specific differences.

### Participants

Volunteers were recruited via social media advertisements. Those who expressed interest underwent an eligibility assessment during an in-person screening visit. Inclusion criteria were: a) men and women aged 60 years or older who had not engaged in regular exercise programs in the last 6 months. Exclusion criteria were: a) neurological disease; b) arrhythmia; c) use of orthoses; d) physical limitations preventing the execution of the test or exercise; and e) medical contraindications for engaging in an exercise program. All participants gave their informed consent and were instructed not to start any other organized exercise program during the study. Initially, 32 elderly individuals from the local community were recruited. After applying the inclusion/exclusion criteria, 24 volunteers were divided into two groups of 12 individual each: the self-selected group (SS) and the imposed group (IMP). All participants underwent a four-stage process: familiarization, pre-intervention tests, intervention, and post-intervention tests.

### Familiarization

Participants were familiarized with the correct execution of movements using the equipment utilized in the study, which included the bench press, the knee extension machine, the lat pulldown, and the knee flexion machine. Standardized instructions regarding subjective perceived exertion scales (PSE OMNI-RES 0-10) and the Hardy et al. (12) sensation scale were provided during this stage.

### Pre-intervention tests

Participants attended two non-consecutive laboratory sessions where trained evaluators administered tests. The assessments during the initial visit included: a) timed up and go (TUG); b) five times sit to stand test; c) walking speed; and d) an orientation session with the isokinetic dynamometer to conduct the maximal voluntary isometric contraction (MVIC) using the Biodex Medical Systems Inc. apparatus (USA) to minimize potential learning effects. On the subsequent visit, participants underwent evaluations for MVIC of knee extensors, assessment of muscle architecture, and composition of the vastus lateralis by ultrasound imaging.

### Body composition

Height, in centimeters, was determined using a stadiometer (Sanny^®^, Standard model, Brazil) fixed to the wall and graduated in 0.1 cm. Participants were asked to remove their footwear, stand on the stadiometer's base, distribute their body weight evenly on both feet, and keep their arms relaxed at their sides with palms facing the thighs. The head was aligned with the Frankfurt plane, the heels together lightly touching the vertical edge of the stadiometer. The measurement was taken with the stadiometer's slider touching the highest point of the head while the individual inhaled (13). Body mass, measured in kilograms, was determined using a digital scale (Toledo^®^, model 2096, Brazil) with an accuracy of 0.1 kg. The participant was barefoot and wore light clothing, stood on the center of the scale platform facing away from the scale, in an anatomical position, with body mass evenly distributed on both feet (13).

### Isokinetic muscular strength

The Biodex Multi-Joint System 3 was used to assess the maximum voluntary isometric strength of the knee extensors. Participants were positioned according to the manufacturer's guidelines (14). The testing procedure included three sets of five seconds of maximum isometric contractions of the knee extensor muscles in the dominant leg, with a two-minute break between sets. Participants were verbally encouraged to exert maximal force. Data were collected at 1000 Hz, and analysis of all strength tests was conducted using Biodex System 3 Advantage software, version 3.2.

### Functional capacity

Functional capacity was assessed through a sequence of three standardized tests, each separated by a five-minute interval. Participants did a trial run for the following tests: the five-times sit to stand test, in which the participant has to transfer from a seated position to a standing position using a chair without armrests with their arms crossed in front of their chest five times in the shortest time possible; the timed up and go (TUG), in which participants walked 3 m, maneuvered around a cone at the end of the path, and returned to a seated position at their regular walking pace; and a 10-m walking test at maximum speed, in which participants covered a 14-m distance at their top speed, with only the time taken to traverse the central 10 m measured. The initial and final 2 m were used for acceleration and deceleration phases.

### Muscular architecture and composition.

Muscular architecture and composition were evaluated using ultrasound imaging (Konica Minolta^®^, Sonimage HS1 model, Japan) with a transducer measuring 5 cm in length by 2 cm in width, operating at a frequency of 11 MHz. The vastus lateralis muscle was selected for analysis due to its large cross-sectional area, being among the largest muscles in the human body. Furthermore, among the four quadriceps heads, it is considered the most potent muscle and plays a significant role in generating force during walking (15). Participants were instructed to refrain from any lower limb exercises 48 h prior to the imaging procedure. The participants rested for 20 min in a supine position with the evaluated limb extended and relaxed to allow for fluid accommodation in the body before data collection (15). Participants were instructed to relax their leg muscles as much as possible during measurements. The proximal insertion of the VL (vastus lateralis) muscle was identified and marked on the skin, and axial sections were then marked at 30-mm intervals. Positioned in the axial plane, the transducer was aligned perpendicularly to the VL muscle and moved from the center to the lateral position along a pre-marked template on the skin. Thigh length was considered as the distance between the lateral femoral epicondyle and the greater trochanter. Once the evaluator deemed the image on the monitor satisfactory, it was archived, and proprietary software of the equipment was used to measure in millimeters using a cursor the linear distance between the fat-muscle interface and the muscle-bone interface. For assessment, a conductive water-based gel was applied to the area to be evaluated, and the transducer was positioned without depressing the skin.

### Fascicle length, pennation angle, muscle thickness, and echo intensity

Once the ultrasound images were available, the fascicle length, pennation angle, muscle thickness, and echo intensity were measured using ImageJ software (version 1.5, National Institutes of Health, USA). Calibration of the software relied on a known 1-cm distance within the images provided by the ultrasound measurement tool. The pennation angle (PA) was ascertained by the angle formed between the muscle fascicles and the deep/internal aponeurosis, aligning with the muscle's line of traction. Fascicle length (FL) was measured as the linear distance between the origin of the fascicle at the internal aponeurosis and its insertion at the external aponeurosis. Muscle thickness (MT) serves as an indirect parameter for cross-sectional area and muscle volume, determined by the perpendicular distance between the fat-muscle and muscle-bone interfaces. Echo intensity (EI) was determined according to the study by Caresio et al. (16) by obtaining the average grayscale value of the muscle using ImageJ 1.42q software. EI is determined using a standard grayscale histogram function and is reported as a value between 0 (black) and 255 (white).

### Maximum dynamic strength (1RM)

Forty-eight hours after muscular architecture and composition were obtained, the maximum dynamic muscle strength was assessed using the 1 repetition maximum (1RM) test for the four prescribed exercises. The testing protocol adhered to the procedures outlined by Fatouros et al. (17).

### Intervention

Thirty-six training sessions were conducted with a 48-h interval between each session. All exercises were performed using machines because of their ergonomic positioning and ease of load adjustment. Participants lay on a bench with both feet flat on the ground. Each repetition involved lowering the bar until it touched the chest, then lifting the bar until the elbows were fully extended. Participants sat upright and pulled the bar towards their chest during the concentric phase. They then slowly returned the bar to the starting position during the eccentric phase. Participants were seated at the machine with their ankles secured under a padded lever, adjusted to ensure that the knee joint was aligned with the machine's pivoting point. They flexed their knees by pulling the lever down towards their body. Participants started in a seated position on the machine with their ankles positioned under the padded lever. The concentric phase involved extending the knees by pushing the lever upward, followed by returning to the initial position.

Participants enrolled in the SS intensity intervention were instructed to select a weight allowing them to complete three sets of 10 repetitions using the following instruction: “What weight would you choose to complete three sets of 10 repetitions for this exercise?” Throughout the intervention, participants could modify the loads after completing each set. The researcher regulated the speed of muscle actions by verbal cues, ensuring participants maintained a cadence of roughly 2 s for both the concentric and eccentric phases of the movement. Participants engaged in the imposed intensity intervention were designated as the Imposed Group (IMP) and followed the exercise prescription model recommended by the ACSM. According to this model, sedentary older adults should perform strength exercises using loads ranging between 50 and 70% of their 1RM. Hence, participants in this group initiated the intervention with a load set at 50% of their 1RM, progressively increasing by 10% every four weeks. To monitor strength gains across all groups, a new 1RM test was conducted every 4 weeks. The training parameters, including the number of sets, repetitions, execution speed, and rest intervals were in alignment with the American College of Sports Medicine's training progression guidelines (18). These guidelines recommend novices engage in both single-joint and multi-joint exercises, performing three sets of 8 to 10 repetitions at a moderate execution pace. A two-minute interval between exercises was given to allow individuals to reach the equipment, adjust loads, and position themselves correctly. The sequence of exercises within the sessions mirrored the order observed during the 1RM test. During each exercise, participants completed 10 repetitions. Preceding each training session, participants underwent a targeted warm-up involving 12 repetitions at 30% of their 1RM. The time gap between this specific warm-up and the subsequent exercises in the session was 2 min. The intervals between sessions allowed for a minimum of 48 h and a maximum of 96 h between each session. The rated perceived exertion (RPE) and affective valence scales were administered at the conclusion of each session in random order.

### Post-intervention assessment

Upon the conclusion of the 36 training sessions, participants underwent subsequent testing to reassess functional capacity, isokinetic muscular strength, maximum dynamic strength, and muscular architecture and composition.

### Statistical analysis

Descriptive statistics (means±SD) were conducted to characterize the groups. Data distribution was tested using boxplots and the Shapiro-Wilk test. Group comparisons before and after the intervention were performed using the linear mixed model test. In the case of a significant F value, Bonferroni's *post hoc* test was used to identify specific differences. Additionally, the effect size was measured by Cohen's d, considering d=0.20 as a small effect, d=0.50 as a medium effect, and d=0.80 as a large effect. The significance level was set at P<0.05, and statistical procedures were conducted using JAMOVI software, version 2.3.9.

## Results

### Descriptive analysis

The SS group was composed of 8 women and 4 men (mean age=66.92±6.18 years; mean body mass=68.75±13.46 kg; mean height=1.70±0.09 m) and the IMP group was composed of 8 women and 4 men (mean age=65.33±2.42 years; mean body mass=69.53±9.93 kg; mean height=1.66±0.06 m).

Table 1 shows the participants’ initial anthropometric characteristics. There were no significant differences between the IMP group and the SS group.

**Table 1:** Initial anthropometric characteristics of participants.

	Total (n=24)	SS (n=12)	IMP (n=12)	P
Age (years)	66±4.66	66.92±6.18	65.33±2.42	0.40
Body mass (kg)	72.38±11.29	68.75±13.46	69.53±9.93	0.42
Height (m)	1.65±0.07	1.64±0.10	1.66±0.06	0.48
BMI (kg/m^2^)	26.69±3.84	26.33±3.12	27.05±4.50	0.65

Data are reported as means and SD. Linear mixed model test. SS: self-selected load group; IMP: imposed load group; BMI: body mass index.

### Muscle function

Both groups had a substantial increase in peak torque to body weight ratio (peak TQ/BW) (F (1,22)=54.294, P≤0.01), along with peak torque (F (1,20)=17.820, P≤0.01). No significant interaction was found between groups (Table 2).

**Table 2:** Peak torque to body weight ratio (TQ/BW) and peak torque for dominant knee extensor muscles at baseline and after 12 weeks of training.

	SS (n=12)		P	ES	IMP (n=12)		P	ES
	Baseline	12 weeks			Baseline	12 weeks		
Peak TQ/BW	2.10±0.57	2.44±0.55	0.01*	1.17	2.12±0.47	2.68±0.57	0.02*	0.51
Peak torque	146±28.7	171±44.7	0.04*	0.03	151±40.5	171±53.5	0.03*	0.18

Data are reported as means and SD. *P<0.05; linear mixed model test. SS: self-selected load group; IMP: imposed load group; ES: effect size.

### Maximum dynamic strength (1RM)

**Figure 1:**
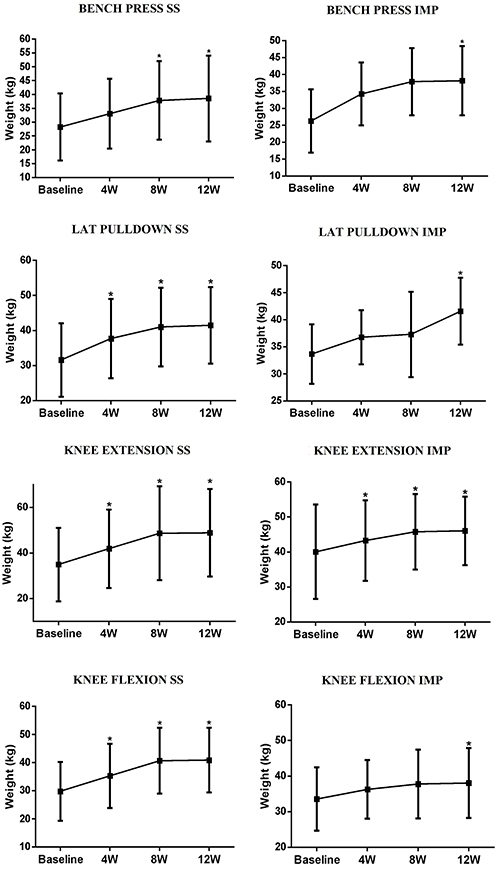
One repetition maximum (1RM) on the bench press, lat pulldown, knee extension, and knee flexion of self-selected group (SS) and imposed (IMP) group. 4W, 8W, and 12W: test after 4, 8, and 12 weeks of intervention, respectively. Data are reported as means and SD. *P<0.05 compared to baseline; linear mixed model test.

Figure 1 displays the results of the 1RM tests. An increase in maximum dynamic strength was observed in both groups after 12 weeks of training in the bench press (F (3,66)=58.97, P<0.01), front pulley (F (3,66)=26.44, P<0.01), knee extension (F (3,66)=36.08, P<0.01), and knee flexor (F (3,66)=51.76, P<0.01) machines. The relative load used by the SS group was 59.7% of 1RM on average in all exercises and the IMP group 60.1% of 1RM on average, with no significant difference between the groups.

### Muscular architecture and composition

Results of muscle architecture and composition are presented in Table 3. MT, FL, PA, and EI did not show significant differences after 12 weeks of training in both groups. No significant interactions were found.

**Table 3:** Muscular architecture and composition at baseline and week 12 of SS group and IMP group.

	SS (n=12)		P	ES	IMP (n=12)		P	ES
	Baseline	12 weeks			Baseline	12 weeks		
E.I	53.46±11.79	51.18±13.26	1.00	0.25	56.03±12.16	53.55±9.43	1.00	0.30
P.A	19.48±7.50	20.11±9.12	0.16	0.53	15.39±2.54	14.50±2.89	1.00	0.59
M.T	1.99±0.40	2.19±0.30	0.44	0.62	2.00±0.40	2.17±0.53	0.97	0.35
F.L	8.14±0.92	8.59±1.31	1.00	0.56	9.09±2.17	9.57±2.40	0.87	0.04

Data are reported as means and SD. Linear mixed model test. SS: self-selected load group; IMP: imposed load group; ES: effect size.

### Rated perceived exertion and affective responses

Table 4 shows the mean values of RPE and affective responses for every 4 weeks. A significant difference was found in the interaction between groups in the last 4 weeks for RPE (F (2,44)=16.47, P≤0.01) and affect (F (2,44)=3.14, P≤0.01).

**Table 4:** Mean values of rated perceived exertion (RPE) and affective response every 4 weeks for both groups.

Weeks	RPE		P	ES	Affective response		P	ES
	SS	IMP			SS	IMP		
1- 4	3.02±1.19	2.63±0.73	0.82	0.18	2.95±0.55	2.48±0.46	0.42	0.25
5-8	3.67±1.09	3.62±1.26	0.99	0.31	2.61±0.59	2.29±0.51	0.79	0.09
8-12	3.23±1.18	5.44±1.21	0.01*	0.50	3.01±0.49	1.76±0.55	0.01*	0.88

Data are reported as means and SD. *P<0.05; linear mixed model test. SS: self-selected load group; IMP: imposed load group; ES: effect size.

### Rate of torque development (RTD)

Table 5 shows the RTD changes. Notably, within the initial 30 ms (RTD 30), a substantial increase was observed after 12 weeks of training in both groups (F (1,22)=122.3674, P<0.01). However, no significant differences were found in the other windows assessed (RTD 50, 100, 200). No significant interactions were found between groups.

**Table 5:** Rate of torque development (RTD) at baseline and after 12 weeks of training.

	SS (n=12)		P	ES	IMP (n=12)		P	ES
	Baseline	12 weeks			Baseline	12 weeks		
RTD 30	288±148	490±191	0.01*	1.58	349±159	524±201	0.01*	0.71
RTD 50	353±188	485±321	0.32	0.54	418±221	500±290	1.0	0.12
RTD 100	370±155	473±355	0.07	0.43	364±274	455±321	0.13	0.25
RTD 200	343±205	399±200	0.06	0.36	301±191	379±299	0.11	0.26

Data are reported as means and SD. *P<0.05; linear mixed model test. SS: self-selected load group; IMP: imposed load group; ES: effect size.

### Functional capacity

Table 6 displays the baseline and 12-week values for TUG, sit-to-stand test, and T10. Significant differences were observed in all tests following the intervention period for both groups: TUG (F (1,22)=49.7310, P<0.01), sit-to-stand (F (1,22)=64.34, P<0.01), and T10 (F (1,22)=63.0992, P<0.01). No significant interactions were found.

**Table 6:** Functional capacity of both groups at baseline and 12 weeks.

	SS (n=12)		P	ES	IMP (n=12)		P	ES
	Baseline	12 weeks			Baseline	12 weeks		
TUG (s)	7.36±0.63	6.15±0.53	0.01*	1.11	7.89±0.72	6.32±0.46	0.03*	0.94
Sit to Stand (s)	10.09±3.32	8.01±2.28	0.01*	1.21	11.01±3.50	10.06±1.08	0.03*	0.87
10 m walking (s)	7.02±2.23	5.01±2.40	0.04*	0.96	9.09±1.57	7.81±0.83	0.03*	0.82

Data are reported as means and SD. *P<0.05; linear mixed model test. SS: self-selected load group; IMP: imposed load group; TUG: timed up and go; ES: effect size.

## Discussion

This study aimed to compare the chronic impact of strength training with self-selected or imposed loads on strength, muscle function, functional capacity, muscular architecture and composition, RPE, and affective responses among the elderly. Both groups had similar improvements in strength and functional capacity. Moreover, the SS group reported lower RPE and higher affective responses compared to the IMP group after 12 weeks. These results suggested a possible influence of load variation and psychophysiological responses, as the IMP group exercised at 70% of 1RM, whereas the SS group used 60% of 1RM during the last four weeks.

The SS group exercised at approximately 60% of their 1RM on average and was able to achieve dynamic and isometric strength gains similar to the IMP group after 12 weeks. Notably, prior studies employing single-session strength exercises have suggested that self-selected loads might not be able to improve muscle strength or cause hypertrophy in physically inactive adults and elderly (10). However, our findings demonstrated that the increase in strength among physically inactive elderly individuals using self-selected loads over a 12-week period mirrors the strength gains observed in various studies that used imposed loads (19,20).

The exercise program in this study aimed to improve both muscle function and functional capacity in elderly individuals. After 12 weeks of resistance training, both groups had notable improvements at RTD 0-30 ms, while no substantial improvement was observed at the 30-50, 50-100, and 100-200 ms phases. Also, both groups had improvements in peak torque. These discoveries are important, as RTD appears more closely associated with daily activities of older adults and more sensitive in identifying immediate and enduring changes in neuromuscular function compared to assessments solely focused on maximal strength (21,22). One possible reason behind the RTD improvements is the improvement in participants' maximum strength, which has a significant correlation with RTD. The study by Andersen and Aagaard (23) demonstrated that an increase in maximal strength had a positive impact on RTD 10-50 ms, similar to the current study. Hence, it is plausible that factors that improve maximal strength (neural drive and cross-sectional muscular area) could have a similar effect on RTD.

However, additional factors might contribute to increased muscle activation during the initial phase of contraction apart from inherent muscular aspects (23). Rapid muscle activation is likely influenced by neural elements such as the firing rate of the motor unit, which could significantly impact the initial contraction phase (first 30-75 ms) (22,24). Studies by Casartelli et al. (25) and Klass et al. (24) have found that neural factors are strongly correlated with contraction initiation. A limitation of the study was the absence of electromyography assessment to evaluate motor unit activation levels.

Both peak TQ/BW and peak torque were significantly improved in both groups following the 12-week intervention. These findings are important as both peak torque and the torque rate development are crucial indicators of functional performance (19,26). Palmer et al. (26) explored the efficacy of knee extensor peak torque and torque rate development in distinguishing the functional status in elderly women capable of walking 550 m in 6 min from those who are not. The results showed that women with higher functionality had greater knee extensor peak torque compared to those with lower functional levels. Similarly, Morcelli et al. (19) reported that knee and hip extensor peak torque had a significantly higher predictive value for gait speed compared to the hip and knee flexors and the ankle plantar flexors.

Similar outcomes were observed in a study by Orssatto et al. (27), which examined the peak torque of knee extensors and flexors in 12 elderly individuals following a three-month progressive resistance training protocol. Conversely, Mair et al. (28) reported moderate and non-significant improvement in knee extensor muscle strength (approximately 9%) after a six-week lower limb training intervention conducted at home. However, they also noted an increase in peak power (approximately 11%) during maximal isometric contraction testing using an isokinetic dynamometer. Discrepancies in results between Mair et al. (28) and the current study might be attributed to the shorter intervention duration, as twice the number of sessions were conducted in the present study.

Both groups improved in every functional capacity test. This is relevant since knee flexor and knee extensor muscles play an important role in daily activities such as walking, climbing stairs, sitting, and standing up from a chair (29). Research indicates that even small increases in muscle strength can lead to functional improvements in older adults, given that muscle strength is a key physiological factor that contributes significantly to the overall functional capacity of the elderly (30).

In this study, the improvements in strength and RTD 0-30 ms potentially contributed to the enhancement in functional capacity, as the sit-to-stand test and 10-m walk time decreased by 21 and 29% in the SS group, and the TUG and sit-to-stand test decreased by 20 and 27% in the IMP group. These findings were similar to the discoveries of Bento et al. (31), who reported higher RTD among non-faller elderly individuals compared to fallers. According to their study, older adults with elevated RTD tend to be better able to regain balance and prevent falls after tripping. Additionally, the research by Casartelli et al. (25) implies that increased TDT 0-75 ms is crucial for elderly individuals to counteract sudden disruptions in postural balance. Hence, increased RTD appears to be a robust predictor of effectiveness of lower-intensity functional tasks.

In the gait speed test, both groups showed improvement after the intervention. Notably, both groups achieved speeds above 1.0 m/s, with recorded values of 1.99 m/s for the SS group and 1.13 m/s for the IMP group. These outcomes are important as speeds below the 1.0 m/s threshold are associated with potential falls, decreased functionality, cognitive impairment, and mortality risk in the elderly (32). These findings closely resonate with previous research such as the studies conducted by Lustosa et al. (33), which demonstrated improved gait speed among older adults following lower limb muscle strengthening programs. Furthermore, Lopopolo et al. (34) reported that a mere 0.1 m/s reduction in gait speed is correlated with a 10% decline in an individual's capacity to perform daily activities. Hence, these discoveries underscore the efficacy of both imposed and self-selected exercise programs as compelling strategies to increase gait speed in older adults, potentially mitigating or delaying age-related functional declines.

Both groups improved their performance in the TUG test from baseline to 12 weeks and they exceeded the suggested performance thresholds for their age. Throughout the study, the IMP and SS groups had average times of 7.10 and 6.75 s, respectively. These results indicated that both groups had significant improvements despite their satisfactory performance at baseline. The observed improvements were likely due to the participants' increased muscle function following the intervention period. Furthermore, these results highlighted the effectiveness of both the prescribed and self-selected exercise load in improving functional capacity and dynamic balance, thereby improving postural stability and decreasing the likelihood of falls, injuries, and decreased independence (35).

Both groups had better performance in the sit-to-stand test following the intervention. Prior research has shown that older adults engaging in resistance training programs improve lower limb muscle strength as assessed by the sit-to-stand test, a phenomenon often linked to the principle of training specificity (36).

Regarding RPE and affective responses, monthly means were compared and the SS group showed lower RPE values (3.23±1.18) and higher affective responses (3.01±0.49) compared to the IMP group (5.44±1.21 and 1.76±0.75, respectively) in the last month of the intervention. These results were intriguing as the percent load of the two groups was similar (60% of 1RM). However, the average load used by the IMP group in the last month was 70% of 1RM while that of the SS group was 59.2% of 1RM. This difference could be one of the factors associated to the significant differences in RPE and affective responses between the groups. Additionally, the RPE in the SS group remained stable over the 12 weeks, ranging between descriptors 3 and 4, which correspond to “easy” and “somewhat easy” perceived effort. This can be explained by the fact that the self-selected load throughout the intervention was probably chosen in such a way that it was perceived as pleasant or less strenuous. Regarding affective responses, both groups perceived the exercise sessions as pleasant throughout the protocol with values between 1 and 3 on the feeling scale, corresponding to a feeling between “fairly good” and “good”.

The findings from this study show that self-selected training load was effective to enhance muscular fitness while being perceived as enjoyable. This is particularly noteworthy since there is evidence indicating that participants' emotional experiences during exercise might predict future engagement in aerobic activities (37). Therefore, given the positive affective responses associated with the self-selected approach, this training method could potentially enhance adherence to resistance training.

Regarding muscular architecture and composition, no significant differences were found between groups. A plausible explanation for this finding could be that the 12-week training duration and the load used were insufficient to induce muscle alterations. This was similar to the studies by Scanlon et al. (38) and Stragier et al. (39) who found that a 6- and 12-week resistance training, respectively, led to increased muscle strength but no changes in the pennation angle of the VL. These outcomes suggest that the increased muscle strength might have stemmed from neural factors such as inter- and intramuscular coordination.

### Limitations

A limitation of the study was that only sedentary elderly individuals were included, which limits the applicability of the results to broader groups (e.g. physically active, frail, or pre-frail elderly). Furthermore, the study did not take into account individual differences such as personality traits and behavioral regulation, which could have influenced perception and affective responses.

### Conclusions

The findings of this study highlighted the effectiveness of resistance training programs with both imposed and self-selected intensity resistance training programs in improving muscle strength and functional capacity among older adults. Notably, the self-selected intensity group showed higher affective responses and lower perceived exertion compared to the imposed intensity group. Such findings provide a basis for new research that considers autonomy and affective responses in the context of resistance training.
